# Surgical Club of South West England

**Published:** 1984-04

**Authors:** 


### Surgical Club of South West England

Meeting held at Weston-super-Mare 21st October 1983

### ROUTINE ENDOSCOPY IN UPPER G. I. BLEEDING

**A. L. Gough, Weston-super-Mare General Hospital**

It has been established that 85–90% of upper G.I. bleeds stop spontaneously, either before or soon after hospital admission, and the value of routine endoscopy in all patients has been questioned.

We admitted 94 patients with well documented bleeding in an eighteen month period, 75% of whom underwent endoscopy within 24 hours. The remaining 25% were not endoscoped either because they required immediate surgery or because they were considered too ill from other medical conditions. Ten patients had operations, 4 of whom died, 2 in the group who had been endoscoped, from associated medical problems, and 2 in the non-endoscopy group, both from bleeding following surgery. The remaining patients were either well at follow-up or had returned to their homes elsewhere in the country.

During the past 10 years there has been a considerable reduction in the operation rate for patients presenting with upper G.I. bleeding. This may partly be explained by the increased association of bleeding with the use of non steroidal anti-inflammatory drugs—40% in this study.

We conclude that routine endoscopy is of value in upper G.I. bleeding as:

(1) An accurate diagnosis may be made in about 95% of patients if endoscoped within 24 hours of admission.
(2) Stigmata likely to indicate re-bleeding may be identified.
(3) If re-bleeding does occur, surgery may be undertaken with the diagnosis known.

### POST-OPERATIVE BLADDER IRRIGATION

**A. Hinchliffe, Weston-super-Mare General Hospital**

In order to prevent or deal with clot retention after prostatectomy the Bristol Urologists developed a closed system of bladder drainage with irrigation. The aim was to avoid bladder syringing and catheter changing which increased the risk of infection (or bacteraemia if infection was already established).

The system, now over 20 years old, depends on the Higginson's syringe and irrigation reservoir to allow intermittent bladder lavage as necessary. We have redesigned it in disposable form using transparent materials including a more resilient bulb than the Higginson's and incorporating a 5 litre drainage bag. No extra dripset or bag is necessary so the cost in use is little more than resterilisable hospital made sets.

Although anaesthetic management and modern endoscopic instruments have improved haemostasis during and after prostatectomy, the system is recommended for:

(1) Open prostatectomy.
(2) TUR's with inadequate haemostasis.
(3) Unexpected post-operative clot retention.
(4) Spontaneous clot retention if there has to be any delay in surgical intervention.

The new Set is available from:
Wesmed Ltd., Units F31–35, Lewis Road, Cardiff.

### REVIEW OF UROTHELIAL TUMOURS AND THE ROLE OF URINARY CYTOLOGY

**M. Singh and A. H. Hinchliffe, Weston-super-Mare General Hospital**

A general survey of 101 patients with urothelial tumours was carried out. The role of urinary cytology in the follow up of urothelial tumours is discussed. The mean age of all patients was 64. Males were predominant with a ratio of 2.75 : 1. A great majority (71%) were heavy smokers. Painless haematuria was the most common symptom. The role of urinary cytology in the follow up of urothelial tumours was confusing and inconclusive. There was a high percentage of false negative results. One interesting group of 14 patients emerged out of this survey, 7 of these were known to have urothelial tumours in the past and during follow up, they were found to have positive urinary cytology but no tumour was found on cystoscopy and I.V.U. The other 7 presented with various urinary symptoms, were found to have positive urinary cytology but no tumour was found on cystoscopy and I.V.U. Four out of these 7 have subsequently been found to have urothelial tumours. Two of these had upper tract tumours and the other 2 had tumours of the urinary bladder.

The important conclusion appears to be that apparently false positive cases with positive urinary cytology and no apparent tumour should never be ignored. Secondly, positive cytology may precede the cystoscopic or radiological evidence or urothelial tumours. Our findings agree with the findings of other authors.

### MALIGNANCY IN WESTON-SUPER-MARE

**M. E. H. Halford, Weston-super-Mare General Hospital**

The South West Regional Cancer Registry reported an increase of 46% of cases registered in the 20 years from 1955–57 to 1975–77. Weston-super-Mare showed the highest rate for the Region. A review of the incidence of histologically proven new cases of carcinoma of bladder, prostate, breast, intestines, skin and melanoma in Weston-super-Mare confirmed a remarkable increase in incidence (except in squamous carcinoma of skin) from 1963 to 1982. Carcinoma of bladder in men had increased from $18.6/10^5$/year to 44.4 (plus 138%) amd in women from $6.9/10^5$/year to 13.5 (plus 96%). Carcinoma of breast had increased by 35%, prostate by 68%, basal cell carcinoma in men by 130% and in women by 170% and malignant melanoma in men by 222% and in women by 45%. The greatest increase in incidence was in carcinoma of bladder in males over 65; in the over 75's it had risen from 57 to $343/10^5$/year, an increase of 500%. The possible causes of these changes in incidence were discussed. Increased radioactivity was not apparently a factor. Heavy smoking in bladder cancer and ultra violet light in skin malignancy were important, but there must be other factors not yet identified.

### SURGICAL MANPOWER AND CAREER STRUCTURE

**David Bolt, lately Chairman BMA Manpower Committee, Honiton**

There is general agreement that improvement in career structure has become urgent. The suggestions currently under discussion with Government are:

(1) Expansion in consultant numbers where there is a real job to be done, as a preliminary to other changes.

(2) The present Senior Registrar numbers would be appropriate to a moderately expanded consultant grade.

(3) Registrar numbers should be trimmed to correspond closely with SR vacancies, with the FRCS essential for appointment.

(4) Possibly some increase in SHO numbers, with the major element of competition for surgical careers taking place between SHO and Registrar grades.

(5) A properly organised system of training for overseas doctors by a sponsorship scheme linked to return home after training. The additional numbers of trainees would be particularly valuable at Registrar level.

The situation is complicated by the current lack of the resources to expand the consultant grade, seen by the profession as the essential preliminary to the other changes.

Reduction in junior hours of work is an additional complication, made necessary by a serious threat of legislation, if the profession cannot achieve it. However, reduction of $\frac{1}{2}$ to $\frac{1}{3}$ rotas depends upon the maintenance of standards of patient care and the final decision is for the consultant concerned.

### HYPOCALCAEMIA AFTER SUB-TOTAL THYROIDECTOMY FOR THYROTOXICOSIS

**N. I. Ramus, Bristol Royal Infirmary**

To protect the recurrent laryngeal nerve during subtotal thyroidectomy for thyrotoxicosis the inferior thyroid artery should be ligated in continuity, laterally in the neck. It has been suggested that this approach results in parathyroid devascularisation and permanent hypocalcaemia and should be abandoned for ligation of the arterial divisions on the surface of the gland. Before this major change in practice was accepted the results from a teaching hospital were reviewed.

Eighty one patients who underwent sub-total thyroidectomy for thyrotoxicosis had a 10% incidence of symptomatic hypocalcaemia (corrected calcium $<2.0$ mmol/L) but only a 1.2% incidence of prolonged hypocalcaemia. In the same patients only 1 had a transient right sided recurrent nerve palsy. Five of the eight symptomatic patients required intravenous calcium postoperatively. Only one of these patients was a routine four vessel ligation, the others were a recurrent thyrotoxicosis and three patients who became hypocalcaemic despite only one inferior thyroid artery being ligated in two cases and neither inferior artery in the other.

These results would seem to confirm the wisdom of lateral ligation of the inferior thyroid artery to protect the recurrent laryngeal nerve. They lend no support to the suggestion that in order to protect parathyroid function this teaching be abandoned in favour of a policy of ligation of the arteries on the surface of the gland.

### DISTURBING LOCAL TRENDS IN ACUTE PANCREATITIS

**A. P. Corfield, M. J. Cooper and R. C. N. Williamson, Bristol Royal Infirmary**

The comprehensive review of acute pancreatitis in Bristol (1950–69) described 590 cases with mortality rates 20.5% (first attack) and 1.5% (subsequent attacks). For the past 8 years 'all' patients have entered prospective clinical trials with a lower mortality rate.

To determine the true natural history of acute pancreatitis a retrospective update (1969–79) revealed 737 admissions by 650 patients: 318 males, 332 females with a mean age of 60 (range 3–94). The incidence has increased from 54 cases/million/year (1961–67) to 73 cases (1969–79). Aetiological factors included gallstones (50%: 130 m, 189 f), alcoholism (8%: 45 m, 6 f), operations (3%) and idiopathic (23%). Incidence of alcohol-related acute pancreatitis has increased progressively over 30 years (1950–54: 2 cases 1975–79: 35 cases). Mortality rates were 19.6% (first attack) and 12.1% (subsequent attacks). Death was commoner over age 60 (102 of 370 patients: 28%) than under age 60 (23 of 268: 9%) $P<0.001$.* Forty-four patients were diagnosed postmortem (32% of all deaths).

There has been an absolute increase in both the incidence and number of deaths from acute pancreatitis in Bristol with an increase in alcohol-related disease. The case mortality of a first attack has remained the same despite modern resuscitative methods and subsequent attacks appear more lethal.

* ($\chi^2 = 35.56$ DF = 1)

### IMPLANTATION RECURRENCE IN COLORECTAL CANCER

**H. C. Umpleby and R. C. N. Williamson, Bristol Royal Infirmary**

Exfoliated colorectal cancer cells are reported to be non-viable and therefore an unlikely cause of suture-line recurrence following resection of colorectal tumours (Rosenberg et al. *Br. J. Surg.* 1978 **65**, 188). The presence and viability of exfoliated tumour cells at 30 proximal and 25 distal sites of intestinal transection were investigated in 30 freshly resected tumour specimens. Irrigation of the resection margins was performed with tissue culture medium and the tumour cells isolated on Nycodenz density centrifugation columns. Viability was determined by trypan blue exclusion and hydrolysis of fluorescein diacetate.

The importance of this finding led us to investigate current surgical practice in the prevention of suture-line recurrence. Forty-eight of 72 surgeons canvassed (67%) routinely use an intraluminal cytotoxic agent. The most popular agents are chlorhexidine-cetrimide preparations (n+14), mercuric perchloride (12), povidone-iodine (7) and water (12); noxythiolin, sodium hypochlorite and silver nitrate are used occasionally. The mean duration of treatment is 2 minutes. When assayed for cytotoxicity against tumour cells, prepared from colorectal carcinomas (n = 10), chlorhexidine-cetrimide (Savlodil) and povidone-iodine (Betadine) were lethal at a wide range of concentrations (5–100% of stock solution). Mercuric perchloride (0.2%) was similarly effective, but up to 20% of tumour cells remained viable after exposure to noxythiolin (Noxyflex) and nearly 30% with water. Chlorhexidine-cetrimide and povidone-iodine are the agents of choice to kill exfoliated colorectal cancer cells.

| | Trypan blue exclusion | | |
|---|---|---|---|
| | Cases viable tumour cells | No. viable tumour cells × $10^5$ median + range) | Median %viability |
| Proximal | 17 | 0.55 (0.25–12.5) | 92.5 |
| Distal | 21 | 1.92 (0.25–22) | 79.3 |

| | Fluorescence (no. cases) | Distance (cms) from tumour (median + range) |
|---|---|---|
| Proximal | 15 | 10.8(4–35) |
| Distal | 21 | 7 5(3–20) |

### THE SOUTH WEST TESTICULAR TUMOUR STUDY GROUP—AN UPDATE

**P. J. B. Smith, Bristol Royal Infirmary**

In the 3 years since it was instituted the South West Testicular Tumour Study Group has established protocols in the Region for the investigation and treatment of testicular tumour. Representatives of all the specialities involved in the management of this disease meet at regular intervals to review these results and to establish new themes of treatment and research. Of particular interest has been the development of tumour marker assay services based on the Radiotherapy Centre at Bristol. This now provides over 120 assays each month—a number which is continuing to increase. All patients with suspected testicular tumour should have such pre-operative assays, as the information obtained is essential for proper treatment and follow-up. The advantages of this local service over a supra-Regional assay are self-evident. Furthermore this pre-operative referral for tumour marker now serves as the formal notification for inclusion in the Register. This absolves the surgeon from any additional paperwork. Unfortunately though there are still patients with testicular tumour not undergoing pre-operative tumour marker assay and hence not being included in the Register. Attempts are in hand to encourage all the surgeons in the Region to support the Register and perform the pre-operative assays.

Chemotherapy and Radiotherapy protocols have been kept under constant review by the Group. There is now a tendency towards a shorter simpler course of cytotoxic drugs with obvious advantages to the patients. There is a review of the position and use of Radiotherapy in the treatment of tumours. Coincidental to this has been the development of interest in the place of abdominal lymphadenectomy for residual bulk disease. Techniques involved in the excision of this secondary tumour are being developed and it is hope, they will be standardised in the Region, possibly using one of two Centres.

The Study Group wishes to thank all those surgeons who have supported it during its formative years, and now that it is established, would encourage any still uncertain to join the Register and admit their patients accordingly.

### CRYPT CELL PRODUCTION RATE IS FASTER IN ALL PHASES OF ULCERATIVE COLITIS COMPARED WITH NORMAL MUCOSA

**A. Allan, Bristol Royal Infirmary**

If an increased colonic cell production rate should occur in patients with ulcerative colitis, this might contribute to the increased incidence of colonic cancer seen in colitic patients. We report in-vitro culture of rectal mucosa from patients with ulcerative colitis, followed by measurement of crypt cell production rate CCPR in the cultured biopsies.

Organ culture of rectal biopsies were carried out for 16 hour followed by a further 3 hour over Vincristine-containing medium. Electron microscopy revealed good preservation of histological architecture during culture. Vincristine arrested dividing cells in metaphase and following crypt microdissection these metaphase figures were counted to derive the CCPR.

Linear accumulation doses of metaphase figures by cultured biopsies was observed ($P<0.001$). Optimal doses of Vincristine to induce metaphase arrest in normal and colitic mucosa were established. Mucosa from colitic patients in histological relapse n = 8, showed a faster CCPR (14.2±s.e. 0.78 cells/hr) than mucosa from colitic patients in remission n = 14, (CCPR 9.78±s.e. 0.48 cells/hr; $P<0.01$). The CCPR of colitic patients in remission was faster than the CCPR of patients with histologically normal mucosa (CCPR 8.58±s.e. 0.36 cells/hr n = 14; $P<0.1$).

The CCPR in mucosa from patients with ulcerative colitis in relapse is significantly faster than the CCPR in mucosa from patients with colitis in remission or normal mucosa. The increased CCPR seen in all phases of the disease may contribute to the increased cancer risk seen in colitic patients.

### LAPAROSCOPY IN THE MANAGEMENT OF OESOPHAGEAL AND GASTRIC CARCINOMA

**A. Shandall* and C. Johnson, Frenchay Hospital, Bristol**

Laparoscopy although well established in gynaecology has gained limited acceptance in general surgery. It is simple, safe and cost-effective in staging and assessing operability. The accuracy rate of liver scintigraphy, ultrasound and CT scanning, single or composite is 80%. Surgery for oesophageal and gastric carcinoma has a high morbidity, mortality and statistically low cure rate. A prospective study of the accuracy of liver scintigraphy, ultrasound scanning and laparoscopy, and their effect on management was undertaken. Accuracy was determined by laparoscopic biopsy, laparotomy and autopsy. Fifty patients were studied: 23 oesophageal carcinoma, 14 gastric carcinoma, 13 suspected disseminated intra-abdominal malignancy. The accuracy was 72, 75.5 and 96% for scintigraphy, ultrasound and laparoscopy respectively, with 10% failed ultrasound due to gas. There was one failed laparoscopy due to adhesions and no morbidity or mortality. Laparoscopy revealed nodal and peritoneal spread in 15 patients. Laparotomy was avoided in 58%, and 74% died in the 18 month follow-up period. A preliminary laparoscopy will obviate the need for laparotomy in inoperable cases and allow better planning for potentially curable surgery. This avoids the morbidity and mortality of an exploratory laparotomy and the discomfort and emotional trauma of these patients with advanced disease with low cure rates.

### FLEXIBLE FIBREOPTIC SIGMOIDOSCOPY IN THE ROUTINE OUTPATIENT CLINIC?

**P. A. Grace and C. D. Collins, Musgrove Park Hospital, Taunton**

Flexible fibreoptic sigmoidoscopy is an extremely useful investigation in the elucidation of large bowel pathology. This study examines the practicality of carrying out this procedure on unprepared bowel in the general surgical outpatients in a District General Hospital and discusses our early experience.

Fifty patients with symptoms of large bowel disease were submitted to flexible fibreoptic sigmoidoscopy. The examination was entirely successful in 38 patients. Of the 12 unsatisfactory examinations 5 (10%) were due to faecal obstruction, 6 to diverticular disease and 1 to carcinomatous stricture. Pathology was detected in 30 patients: Diverticular

Disease 13, Carcinoma 3, Adenoma 3, Colitis/Proctitis 8, Solitary Rectal Ulcer 1 and Haemorrhoids 2. Although we did not prospectively compare flexible fibreoptic sigmoidoscopy against other modalities of investigation we estimate that useful information was gained in 14 patients which would not have been obtained otherwise.

We suggest that the advantages of flexible fibreoptic sigmoidoscopy include: early diagnosis of pathology, assessment of the 'doubtful sigmoid', patient comfort and reduction in barium enemas. The disadvantages are: cost (initial outlay of £3000), time (10–30 mins for examination and recycling of equipment), space, skill and training of nursing staff, small biopsies.

In summary we believe that flexible fibreoptic sigmoidoscopy can be used effectively in the unprepared bowel in the general surgical outpatient clinic giving an impressive yield of pathology leading to early definitive treatment and a reduction in requests for barium enemas.

The last 3 papers were selected out of 11 entrants for the S.W. Surgical prize.

